# Widely Targeted Metabolomics Reveals Dynamic Secondary Metabolite Accumulation and Antioxidant Biomarkers Across Ripening Stages of *Ziziphus jujuba* cv. ‘Junzao’ Fruit

**DOI:** 10.3390/antiox15081038

**Published:** 2026-08-20

**Authors:** Yahui Yan, Chaoming Zhang, Yongxia Tao, Zuoshan Feng

**Affiliations:** 1 School of Food Science and Pharmacy, Xinjiang Agricultural University, Urumqi 830052, China; 2 Postdoctoral Station of Horticulture, Xinjiang Agricultural University, Urumqi 830052, China

**Keywords:** *Ziziphus jujuba* Mill. cv. ‘Junzao’ fruits, harvest times, widely targeted metabolomics, secondary metabolites, radical-scavenging capacity

## Abstract

Fruit ripening is accompanied by extensive reprogramming of secondary metabolism, which determines the antioxidant value of medicine-food homologous fruits. *Ziziphus jujuba* Mill. cv. ‘Junzao’ (Junzao) is a high-quality cultivar rich in bioactive compounds, yet its stage-dependent metabolite accumulation and the corresponding antioxidant capacity remain poorly resolved. In this study, widely targeted metabolomics was combined with the quantification of total phenolic (TPC), total flavonoid (TFC), and total triterpenoid (TTC) contents and with 1,1-diphenyl-2-picrylhydrazyl radical (DPPH) and 2,2′-azino-bis(3-ethylbenzothiazoline-6-sulfonic acid) radical (ABTS) radical-scavenging assays to profile Junzao fruits at five developmental stages. A total of 2388 secondary metabolites were identified, with flavonoids and terpenoids representing the major classes. TPC and TFC were highest at the immature YG (young-fruit) stage, whereas TTC peaked at the BS (white-ripe) stage; all three decreased during subsequent ripening, consistent with the stronger DPPH and ABTS radical-scavenging activities observed in early-stage fruits. Multivariate analyses revealed distinct metabolic profiles among developmental stages, and 2111 differentially accumulated metabolites (DAMs) were identified. K-means clustering resolved nine temporal accumulation patterns, and Kyoto Encyclopedia of Genes and Genomes (KEGG) pathway enrichment indicated dynamic regulation of flavonoid biosynthesis, phenylpropanoid metabolism, and triterpenoid-related pathways. Spearman correlation analyses further identified ten metabolites, comprising six flavonoids, three triterpenes, and one phenolic acid, that were strongly associated with antioxidant capacity (|r| ≥ 0.5, *p* < 0.05), highlighting their potential as biomarkers for quality evaluation. Overall, immature Junzao fruits exhibited superior antioxidant capacity, supporting their promise as functional-food ingredients and providing a basis for stage-specific harvesting and utilization.

## 1. Introduction

Oxidative stress, which arises from an imbalance between reactive oxygen species (ROS) and endogenous antioxidant defenses, is a key driver in the onset and progression of numerous chronic diseases, including cardiovascular disorders, diabetes, neurodegeneration, and certain cancers [1,2,3]. Because synthetic antioxidants face persistent consumer and regulatory concerns, dietary antioxidants of plant origin from medicine-food homologous resources are attracting increasing interest as safe and sustainable alternatives for functional-food development [4,5].

Flavonoids and triterpenes are the two major categories of plant-derived antioxidants with both antioxidant and metabolic regulatory properties, making them attractive constituents for antioxidant-oriented functional foods [6,7]. Flavonoids such as quercetin and diosmetin exert hepatoprotective effects by directly scavenging ROS, chelating transition metal ions, activating the Nrf2/Keap1 antioxidant pathway, and suppressing NF-κB-driven inflammation [8,9]. Triterpenoids, including oleanolic acid and ursolic acid, regulate lipid metabolism, improve mitochondrial function, and enhance insulin sensitivity, thereby alleviating hepatic steatosis [10,11]. *Ziziphus jujuba* Mill., honored as the “king of fruits” and classified as a superior-grade medicinal material in Shennong Bencao Jing [12], is a well-established medicine-food homologous resource rich in polyphenols, flavonoids, cyclic nucleotides, triterpenic acids, polysaccharides, and saponins, and exhibits antioxidant, anti-inflammatory, immunomodulatory, neuroprotective, and hepatoprotective activities [13,14,15]. *Ziziphus jujuba* Mill. cv. ‘Junzao’ (Junzao), a high-quality cultivar native to Xinjiang, is valued for its large fruit size and rich phytochemical composition [16]. Junzao accumulates substantial amounts of rutin, epicatechin, catechin, betulinic acid, and oleanolic acid, making it a promising candidate for exploring preventive strategies antioxidant. Nevertheless, research on Junzao has focused predominantly on mature fruits or crude extracts, with limited systematic comparison across developmental stages.

Fruit ripening involves profound secondary metabolic reprogramming [17]. Contrary to the conventional view that “ripe fruits are the most nutritious”, recent plant metabolomic studies increasingly demonstrate that many pharmacologically relevant flavonoids and triterpenoids reach peak abundance at immature or early developmental stages [18,19]. As ripening progresses, metabolic flux shifts toward carbohydrate accumulation and flavor compound synthesis, leading to the degradation or modification of these early-accumulated antioxidants [20]. This stage-specific accumulation pattern is closely associated with plant defense mechanisms. For instance, immature hawthorn and unripe citrus accumulate high levels of bioactive compounds [18,21]. Widely targeted metabolomics combines the broad detection scope of untargeted profiling with the sensitivity and accuracy of targeted analysis, enabling thousands of metabolites to be detected and relatively quantified in a single run, thereby resolving their spatiotemporal dynamics [22,23,24]. Therefore, tracking the dynamic metabolic trajectory during fruit development is crucial for determining the optimal harvest time for targeted bioactive compounds.

Despite the recognized nutritional value of Junzao, its current utilization depends almost exclusively on fully ripe fruits, whereas immature Junzao fruits are largely overlooked or discarded as agricultural waste [25,26,27]. The lack of high-coverage metabolite atlas spanning the full developmental trajectory of Junzao fruit, together with insufficient understanding of the stage-dependent relationship between metabolite accumulation and antioxidant capacity, severely hampers scientific decision-making for precision harvesting and by-product valorization. To address these gaps, the present study integrated metabolomics, spectrophotometric determination of phenolic, flavonoid, and triterpenoid contents and with DPPH and ABTS radical-scavenging assays to map the temporal trajectories of secondary metabolites across five developmental stages of Junzao and to identify the developmental window associated with the highest antioxidant capacity. This work provides a scientific foundation for stage-specific harvesting and for the valorization of immature fruits as functional-food ingredients and natural-antioxidant sources.

## 2. Materials and Methods

### 2.1. Plant Materials and Sample Collection

**Figure 1:**
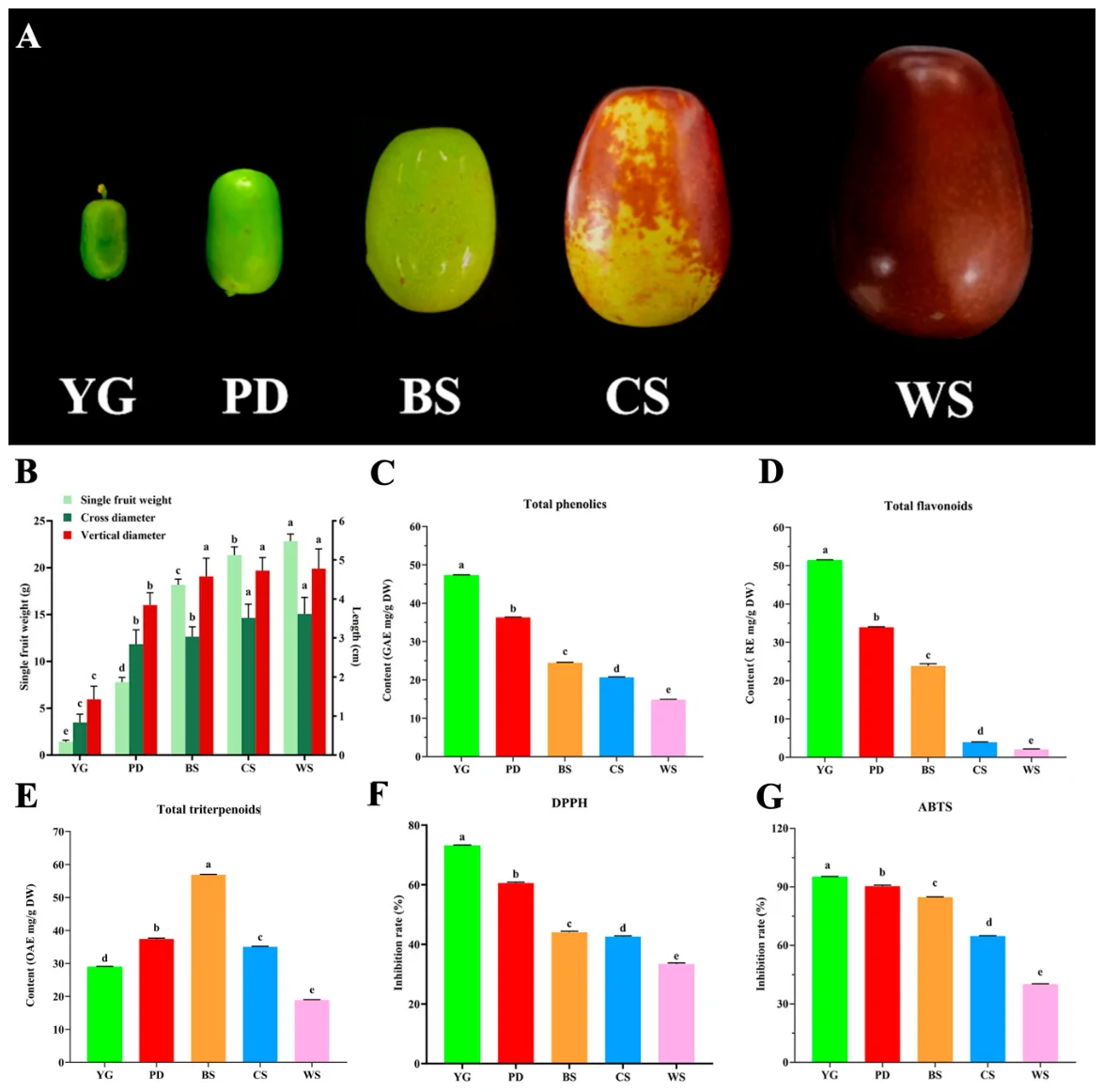
Morphological characteristics, bioactive compound contents, and antioxidant capacities of *Ziziphus jujuba* Mill. cv. ‘Junzao’ (Junzao) fruits. (**A**) Different developmental stages of Junzao fruit: young-fruit stage (YG), expansion stage (PD), white-ripe stage (BS), crispy-ripe stage (CS), and full-ripe stage (WS). (**B**) Fruit weight, transverse diameter, and longitudinal diameter. (**C**) Total phenolic content (TPC). (**D**) Total flavonoid content (TFC). (**E**) Total triterpenoid content (TTC). (**F**) DPPH (1,1-Diphenyl-2-picrylhydrazyl) radical-scavenging activity. (**G**) ABTS [2,2′-azino-bis(3-ethylbenzothiazoline-6-sulfonic acid)] radical-scavenging activity. The different lowercase letters (a, b, c, d, e) above the bar chart indicate significant differences between groups at the *p* < 0.05 level.

Junzao fruits were hand-harvested from the jujube germplasm resource nursery located in Maigaiti County, Kashgar, Xinjiang, China (N38.99°, E77.67°) during the 2023 growing season. Five developmental stages were defined based on days after flowering and morphological characteristics: 28 June (YG, young-fruit stage), 20 July (PD, expansion stage), 14 August (BS, white-ripe stage), 9 September (CS, crispy-ripe stage), and 2 October (WS, full-ripe stage) (Figure 1A). At each developmental stage, fruits of uniform size and free from visible defects or disease were randomly harvested from five-year-old Junzao trees grown under the same climatic conditions and management practices. For both physiological and metabolomic analyses, three biological replicates were prepared for each stage, with each replicate comprising 15–20 fruits. Fresh samples from the five ripening stages were washed, cut into pieces, pitted, immediately frozen in liquid nitrogen, and dried in a vacuum freeze dryer (SCIENTZ-10N/C, Ningbo Xinzhi Biotechnology Co., Ltd., Ningbo, China) for 96 h. The dried samples were ground to a fine, homogeneous powder (100-mesh) using a high-speed universal crusher (FW-10, Beijing Yongguangming Medical Instrument Co., Ltd., Beijing, China), and stored in a −80 °C ultra-low-temperature freezer (DW-HL529BP, Zhongke Meiling Cryogenic Technology Co., Ltd., Hefei, China) until further analysis.

### 2.2. Reagents

Sodium nitrite, sodium hydroxide, aluminum nitrate, and glacial acetic acid were purchased from Tianjin Xinbot Chemical Co., Ltd. (Tianjin, China). Methanol, formic acid, and sodium carbonate were purchased from Tianjin Zhiyuan Chemical Reagents Co., Ltd. (Tianjin, China). Folin–Ciocalteu reagent was purchased from Solarbio Science & Technology Co., Ltd. (Beijing, China), and perchloric acid was purchased from Urumqi Baihe Dazheng Instrument Equipment Co., Ltd. (Urumqi, China). All of the above reagents were of analytical-grade. Rutin (98%), gallic acid (98%), vanillin (99.5%), 1,1-diphenyl-2-picrylhydrazyl radical (DPPH) and 2,2′-azino-bis(3-ethylbenzothiazoline-6-sulfonic acid) (ABTS) were purchased from Shanghai Yuanye Biotechnology Co., Ltd. (Shanghai, China). HPLC-grade formic acid was purchased from Shanghai Aladdin Biochemical Technology Co., Ltd. (Shanghai, China). HPLC-grade methanol and acetonitrile were purchased from Merck (Darmstadt, Germany).

### 2.3. Determination of Fruit Phenotypic Characteristics

Single-fruit weight was measured using an electronic analytical balance (MS105DM, Mettler Toledo, Zurich, Switzerland). Fruit longitudinal and transverse diameters were measured using a digital vernier caliper (150 mm, Shanghai Chixi Measuring Tools Co., Ltd., Shanghai, China). For each stage, 20 fruits were measured, and the mean values were calculated.

### 2.4. Determination of Bioactive Compounds and Antioxidant Activity

#### 2.4.1. Extraction of Bioactive Compounds

The extraction of phenolic compounds, flavonoids, and triterpenoids was performed according to a previously reported method with minor modifications [28]. Briefly, 1.0 g of freeze-dried powder was extracted with 40 mL of acidified aqueous methanol (methanol:water:formic acid = 50:49:1, *v*/*v*/*v*) by ultrasonication (KQ-400DE, Kunshan Ultrasonic Instrument Co., Ltd., Suzhou, China) at room temperature for 30 min. The extract was centrifuged (4K15, Sigma Laboratory Centrifuges, Inc., Osterode am Harz, Germany) at 12,000 rpm for 15 min at 4 °C, and the supernatant was collected and brought to 50 mL with the same acidified aqueous methanol for subsequent assays.

#### 2.4.2. Total Phenolic Content (TPC)

Total phenolic content was determined using the Folin–Ciocalteu method, as described previously with slight modifications [28]. Briefly, 1 mL of extract was mixed with 0.5 mL of Folin–Ciocalteu reagent and incubated for 8 min. Then, 1.5 mL of 20% (*w*/*v*) sodium carbonate solution was added, and the mixture was incubated at room temperature for 24 h in the dark. The absorbance was measured at 765 nm using a UV-Vis spectrophotometer (TU-1810, Beijing Puxi General Instrument Co., Ltd., Beijing, China). Results were expressed as milligrams of gallic acid equivalent (GAE) per gram of dry weight (mg GAE/g DW). The standard calibration curve (y = 0.0227x + 0.0797) was established using gallic acid, yielding an R^2^ of 0.9994 over the range of 3.125–50 µg/mL.

#### 2.4.3. Total Flavonoid Content (TFC)

Total flavonoid content was determined using the aluminum nitrate-sodium nitrite colorimetric method with slight modification [29]. Briefly, 1 mL of extract were mixed with 0.3 mL of 5% NaNO_2_ and allowed to react for 6 min. Subsequently, 0.3 mL of 10% Al(NO_3_)_3_ was added, followed by incubation for 6 min. Then, 4 mL of 5% NaOH and 4.4 mL of 80% methanol were added, and the absorbance was recorded at 510 nm. Results were expressed as milligrams of rutin equivalent (RE) per gram of dry weight (mg RE/g DW). The standard calibration curve (y = 1.1313x + 0.0449) was established using rutin, yielding an R^2^ of 0.9992 over the range of 0.05–1.0 mg/mL.

#### 2.4.4. Total Triterpenoid Content (TTC)

Total triterpenoid content was measured using the vanillin-glacial acetic acid colorimetric method with slight modification [30]. Briefly, 1 mL of sample extract was evaporated to dryness in a 90 °C water bath and then reacted with 0.2 mL of 5% (*w*/*v*) vanillin-glacial acetic acid solution (5 g of vanillin in 100 mL of glacial acetic acid), and 0.8 mL of perchloric acid at 60 °C for 20 min. After incubation, the mixture was rapidly cooled on ice and diluted with 5 mL of glacial acetic acid. The absorbance was measured at 548 nm, and the results were expressed as milligrams of oleanolic acid equivalent (OAE) per gram of dry weight (mg OAE/g DW). The standard calibration curve (y = 0.0102x + 0.0484) was established using oleanolic acid, yielding an R^2^ of 0.9993 over the range of 3.125–100 µg/mL.

#### 2.4.5. Determination of Antioxidant Activities

DPPH and ABTS radical-scavenging activity were assessed according to a previously described method with slight modification [31]. Briefly, 2 mL of extract was mixed with 2 mL of 0.2 mmol/L DPPH in ethanol. The mixture was incubated in the dark at room temperature for 30 min, and the absorbance was measured at 517 nm. The ABTS working solution was prepared by reacting 7 mmol/L ABTS with 2.45 mmol/L potassium persulfate (1:1, *v*/*v*) and incubating the mixture in the dark for 16 h. The working solution was then diluted with phosphate buffer (pH 7.4) to achieve an absorbance of 0.70 ± 0.02 at 734 nm. Subsequently, 1 mL of extract was mixed with 4 mL ABTS working solution and incubated for 10 min in the dark. The absorbance was recorded at 734 nm. Results were expressed as *inhibition rate* (%) and calculated using the following formula:

$$Inhibition\;rate\;(\%)=(1-\frac{A_{1}-A_{2}}{A_{0}})\times 100$$

where *A*_0_ is the absorbance of the mixed solution containing the corresponding solvent and DPPH/ABTS solution; *A*_1_ is the absorbance of the mixed solution containing diluted extract and DPPH/ABTS solution; and *A*_2_ is the absorbance of the mixed solution containing diluted extract and the corresponding solvent.

### 2.5. Widely Targeted Metabolomics Analysis

#### 2.5.1. Sample Preparation and Metabolite Extraction

Freeze-dried jujube fruit samples were ground to a fine powder using a grinding instrument (MM 400, Retsch, Haan, Germany) with zirconia beads for 1.5 min at 30 Hz. Approximately 30 mg of powder was weighed and extracted with 1.5 mL of 70% methanol (*v*/*v*) containing 0.25 mg/L lidocaine as internal standard [32]. The samples were vortexed for 30 s every 30 min for a total of six times. After centrifugation at 12,000 rpm for 3 min at 4 °C, the supernatant was filtered through a 0.22 μm PTFE membrane and stored in injection vials for UPLC-MS/MS analysis. Quality control (QC) samples were prepared by pooling equal aliquots from all individual samples to monitor the reproducibility of the analytical process.

#### 2.5.2. UPLC-MS/MS Analysis

Metabolomics analysis was performed using an ultra-high-performance liquid-chromatography system (UPLC ExionLC™ AD, Applied Biosystems Sciex, Foster City, CA, USA) coupled with a tandem mass spectrometer (Applied Biosystems Sciex Triple TOF 6600 and 4500 QTRAP). Chromatographic separation was achieved on an Agilent SB-C18 column (1.8 μm particle size, 2.1 × 100 mm, Agilent Technologies, Inc., Milford, CT, USA) maintained at 40 °C. The mobile phase consisted of solvent A (0.1% formic acid in water) and solvent B (0.1% formic acid in acetonitrile). The gradient elution program was as follows: 0–9 min, 95–5% A; 9–10 min, 5% A; 10–11.1 min, 5–95% A; 11.1–14 min, 95% A. The flow rate was 0.35 mL/min, and the injection volume was 2 μL.

The mass spectrometer was operated in both positive and negative electrospray ionization (ESI) modes. The ESI source parameters were set as follows: ion source temperature, 500 °C; ion spray voltage, 5500 V in positive mode and −4500 V in negative mode; curtain gas, 25 psi; ion source gas I, 50 psi; ion source gas II, 60 psi; and collision gas, high. Data acquisition was performed in multiple reaction monitoring (MRM) mode based on the Metware database (Metware Biotechnology Co., Ltd., Wuhan, China).

### 2.6. Metabolomics Data Processing and Multivariate Statistical Analysis

Raw data were processed using Analyst 1.6.3 software (AB SCIEX, Framingham, MA, USA) for peak detection, alignment, and integration. Metabolite peak areas were normalized and log_2_-transformed prior to statistical analysis. Metabolite identification confidence was classified into three levels, with Level 1 representing the highest confidence, followed by Levels 2 and 3 in descending order of annotation accuracy. Hierarchical clustering analysis (HCA) was performed using Euclidean distance and average linkage methods with Z-score normalized data to visualize metabolite accumulation patterns. Principal component analysis (PCA) was conducted to assess overall metabolic variations among the different developmental stages. Orthogonal partial least squares discriminant analysis (OPLS-DA) was performed to identify metabolites contributing to group separation. Differentially accumulated metabolites (DAMs) were identified based on variable importance in projection (VIP) ≥ 1 from the OPLS-DA model, combined with |log_2_fold change| ≥ 1, and *p* < 0.05 from univariate statistical analysis [33]. K-means clustering was applied to group DAMs according to their dynamic changes during fruit development. All multivariate analyses were performed using R software (version 4.2.0). Kyoto Encyclopedia of Genes and Genomes (KEGG) pathway enrichment analyses and Gene Ontology (GO) functional annotation for DAMs were performed using the Metware Cloud platform (Metware Biotechnology Co., Ltd., Wuhan, China).

### 2.7. Statistical Analysis

All physiological measurements were performed with three biological replicates, and results were expressed as mean ± standard deviation (SD). One-way analysis of variance (ANOVA), followed by Duncan’s multiple range test, was performed to determine significant differences among developmental stages at *p* < 0.05. Statistical analyses were conducted using SPSS 26.0 software (IBM Corp., Armonk, NY, USA). Figures were generated using GraphPad Prism 9.0 (GraphPad Software, San Diego, CA, USA) and R software (version 4.2.0). Heatmaps, venn diagrams, and other visualizations were created using the Metware Cloud platform and R packages.

## 3. Results

### 3.1. Morphological Characterization of Junzao Fruits During Development

The morphological characteristics of Junzao fruits at different developmental stages are shown in Figure 1A. Fruit color transitioned from green (YG-PD) to yellow-green (BS) and ultimately to deep red (WS), indicating the progressive degradation of chlorophyll and accumulation of anthocyanins during ripening. Distinct morphological changes were observed throughout fruit development. Fruit size increased progressively, with the longitudinal diameter expanding from 1.41 ± 0.30 cm (YG) to 4.86 ± 0.45 cm (WS), and the transverse diameter increasing from 0.81 ± 0.24 cm to 3.70 ± 0.43 cm. Correspondingly, single-fruit weight increased substantially from 1.42 ± 0.17 g (YG) to 26.14 ± 0.92 g (WS) (Figure 1B).

### 3.2. Dynamic Changes in TPC, TFC, TTC and Antioxidant Activities at Different Fruit Growth Stages

Both TPC and TFC exhibited a similar and consistent declining trend as Junzao fruit matured (Figure 1C,D). The highest levels were recorded at the earliest stage (YG), with TPC and TFC reaching 47.30 ± 0.11 mg GAE/g DW and 51.40 ± 0.13 mg RE/g DW, respectively. These values progressively decreased throughout development, reaching their lowest levels at the WS stage, with TPC and TFC values of 14.84 ± 0.11 mg GAE/g DW and 2.04 ± 0.12 mg RE/g DW, respectively. In contrast, TTC displayed a different trend (Figure 1E), gradually increasing from 28.96 ± 0.25 mg OAE/g DW at the YG stages and peaking at 56.86 ± 0.15 mg OAE/g DW at the BS stage. Subsequently, as the fruit ripened, TTC gradually decreased, eventually reaching 18.92 ± 0.18 mg OAE/g DW at the WS stage.

The antioxidant capacities of Junzao fruit at different developmental stages were evaluated using DPPH and ABTS radical-scavenging activity. DPPH and ABTS radical-scavenging capacity reached their maximum values at the YG stage (73.17 ± 0.19%, 95.07 ± 0.29%, respectively) and then declined sharply as the fruit ripened, decreasing to 33.50 ± 0.29% and 40.11 ± 0.14%, respectively, at the WS stage (Figure 1F,G). These results indicated that young and developing fruits possessed stronger antioxidant activities than fully ripe fruits, consistent with their higher phenolics and flavonoid contents.

### 3.3. Metabolite Profiling and Multivariate Analysis of Junzao Fruits

**Figure 2:**
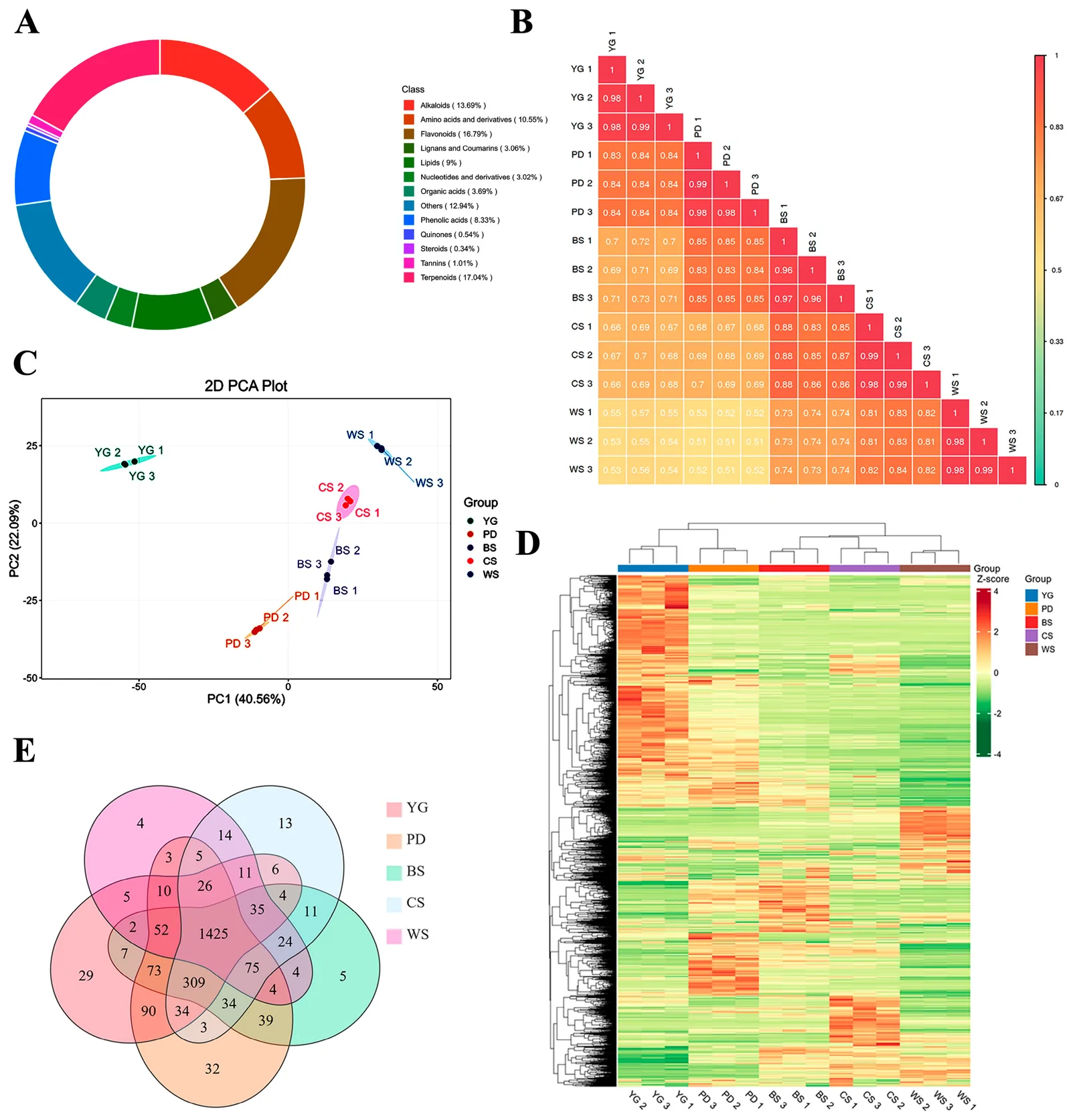
Analytical overview of metabolites detected in Junzao fruits at five developmental stages. (**A**) Classification and proportion of secondary metabolites identified across all samples. (**B**) Pearson correlation analysis. Color intensity represents the correlation coefficient (r), with red indicating a high positive correlation. (**C**) Principal component analysis (PCA) score plot showing the distribution of samples across developmental stages. (**D**) Hierarchical clustering analysis (HCA) heatmap of secondary metabolites. Red indicates relatively high abundance, whereas green indicates relatively low abundance. (**E**) Venn diagram showing the distribution of shared and unique metabolites among the five developmental stages.

To comprehensively characterize metabolic changes during fruit development, widely targeted metabolomics analysis was performed on Junzao fruits at five growth stages. The total ion chromatograms of the QC samples demonstrated excellent reproducibility and stability of the analytical method (Figure S1). A total of 2388 secondary metabolites were identified across all samples, and classified into thirteen major categories: terpenoids (407, 17.04%), flavonoids (401, 16.79%), alkaloids (327, 13.69%), amino acids and derivatives (252, 10.55%), lipids (215, 9.00%), phenolic acids (199, 8.33%), organic acids (88, 3.69%), lignans and coumarins (73, 3.06%), nucleotides and derivatives (72, 3.02%), tannins (24, 1.01%), quinones (13, 0.54%), steroids (8, 0.34%), and others (309, 12.94%) (Figure 2A; Table S1). Among the identified secondary metabolites, flavonoids showed the highest abundance in the fruits and were mainly composed of flavonols (143) and flavones (79). Terpenoids represented the second major category, mainly including triterpenes (138) and triterpene saponins (86).

Pearson correlation analysis was performed to evaluate the relationships among samples (Figure 2B). High correlation coefficients (r > 0.90) were observed among biological replicates within each developmental stage, confirming the reliability of the metabolomics data. PCA was conducted to visualize the overall metabolic variations among different developmental stages (Figure 2C). In the PCA score plot, the first two principal components, PC1 and PC2, explained 62.65% of the total variance, with PC1 contributing 40.56% and PC2 contributing 22.09%. The results showed clear separation among the five developmental stages, while the three biological replicates from the same stage clustered closely together.

In addition, HCA was performed using Euclidean distance and the average linkage method after Z-score normalization to visualize the accumulation patterns of all detected metabolites (Figure 2D). The heatmap revealed stage-specific metabolite accumulation patterns, with distinct clusters corresponding to early (YG, PD), middle (BS, CS), and late (WS) developmental stages, which were generally consistent with the PCA results. Furthermore, venn diagram analysis showed that 1425 metabolites were commonly detected across all five stages, whereas 29, 32, 5, 13, and 4 metabolites were specifically identified in YG, PD, BS, CS, and WS stages, respectively (Figure 2E, Table S2). These results indicated that although most metabolites were present throughout fruit development, certain stage-specific metabolites may contribute to the unique characteristics of each developmental phase.

### 3.4. OPLS-DA Analysis of Metabolic Differences Between Adjacent Developmental Stages

**Figure 3:**
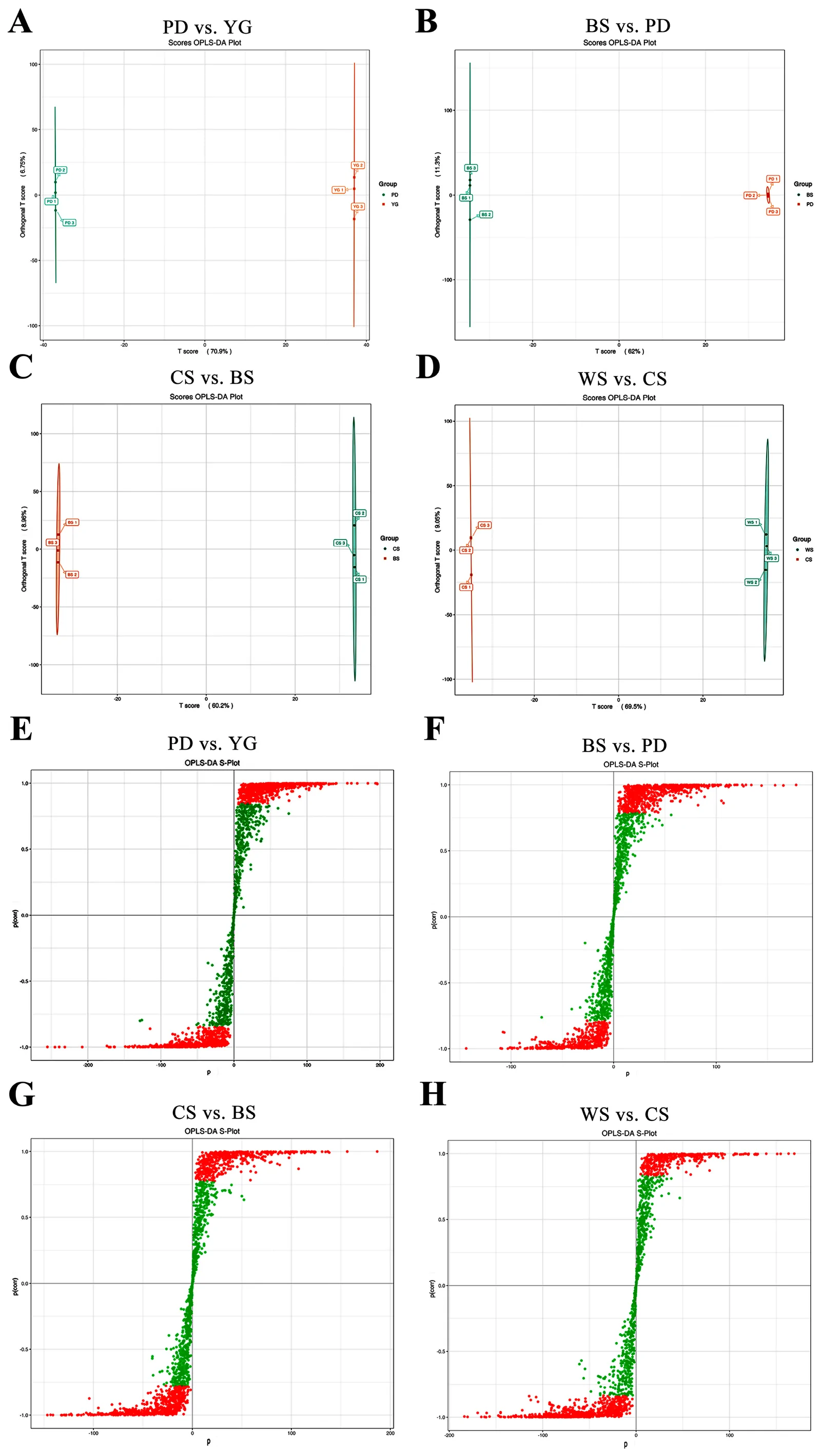
Orthogonal partial least squares discriminant analysis (OPLS-DA) of Junzao fruits in different pairwise comparisons. (**A**–**D**) OPLS-DA score plots showing the separation between two consecutive developmental stages: (**A**) PD vs. YG, (**B**) BS vs. PD, (**C**) CS vs. BS, and (**D**) WS vs. CS. (**E**–**H**) S-plot models corresponding to each pairwise comparison: (**E**) PD vs. YG, (**F**) BS vs. PD, (**G**) CS vs. BS, and (**H**) WS vs. CS. Red dots indicate metabolites with VIP values greater than 1, whereas green dots indicate metabolites with VIP values less than or equal to 1.

To further identify metabolic differences between adjacent developmental stages, OPLS-DA was performed for four pairwise comparisons: PD vs. YG, BS vs. PD, CS vs. BS, and WS vs. CS. All OPLS-DA models exhibited excellent goodness of fit and predictive ability, and permutation tests with 200 iterations were performed for validation: PD vs. YG (R^2^X = 0.777, R^2^Y = 1, Q^2^ = 0.991), BS vs. PD (R^2^X = 0.732, R^2^Y = 1, Q^2^ = 0.975), CS vs. BS (R^2^X = 0.692, R^2^Y = 1, Q^2^ = 0.976), and WS vs. CS (R^2^X = 0.786, R^2^Y = 1, Q^2^ = 0.988) (Figure S2). The combination of high R^2^Y and Q^2^ values, together with successful permutation validation, demonstrated that the OPLS-DA models were robust, with no evidence of overfitting, for identifying DAMs between developmental stages. The OPLS-DA score plots showed clear separation between comparison groups in each model, indicating significant metabolic differences between adjacent stages (Figure 3A–D). The S-plots derived from the OPLS-DA models were used to visualize the contribution and reliability of each variable in discriminating between two groups (Figure 3E–H). Variables positioned far from the origin, particularly in the top-right and bottom-left corners, were considered the most influential for group separation. These variables exhibited both high covariance and high correlation with the predictive component.

**Figure 4:**
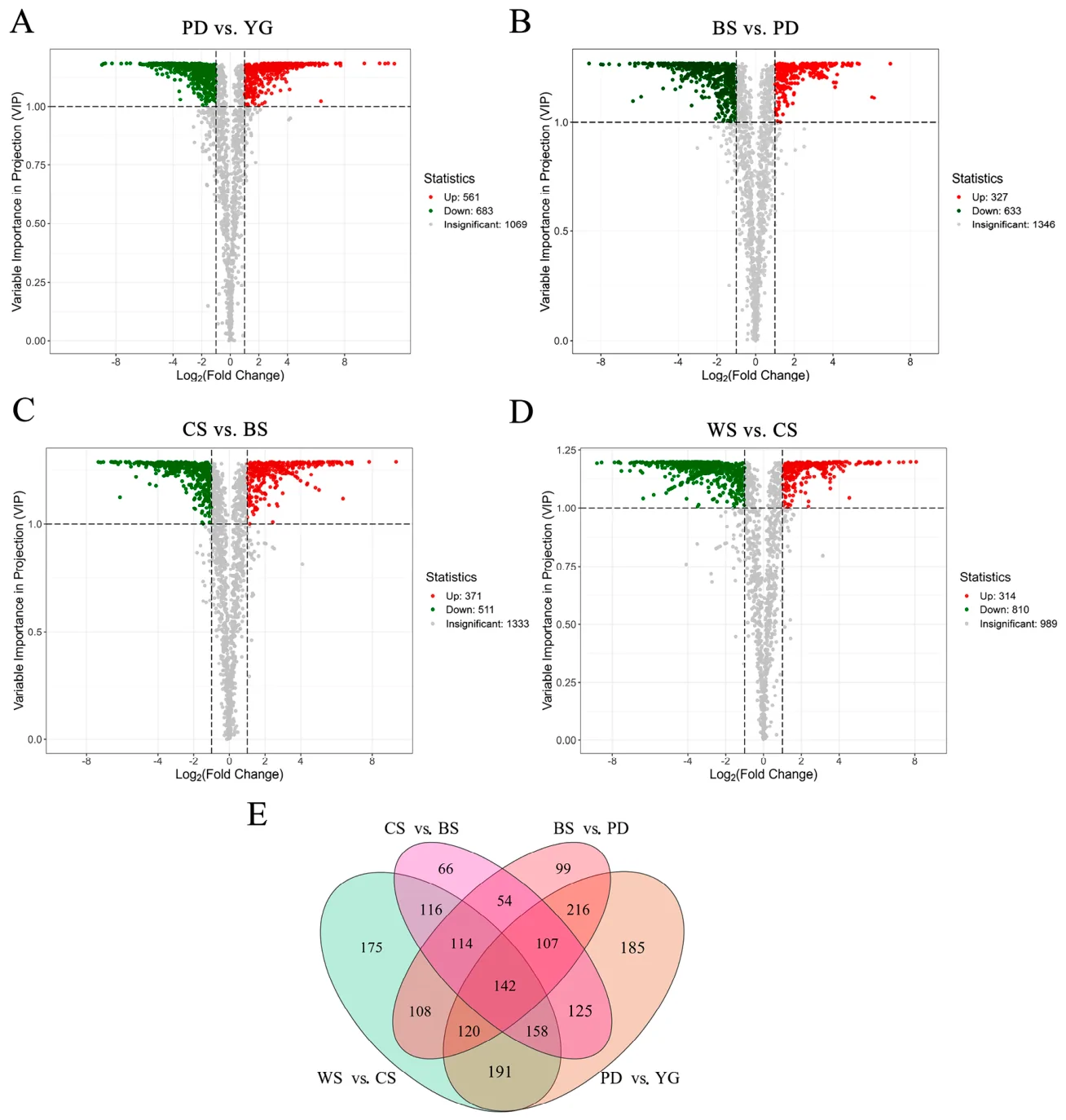
Differential metabolite analysis of Junzao fruits in different pairwise comparisons. (**A**–**D**) Volcano plots showing differentially accumulated metabolites (DAMs) between consecutive developmental stages: (**A**) PD vs. YG, (**B**) BS vs. PD, (**C**) CS vs. BS, and (**D**) WS vs. CS. Red dots indicate significantly upregulated metabolites, green dots indicate significantly downregulated metabolites, and gray dots indicate metabolites with no significant difference. (**E**) Venn diagram showing the number of shared and specific DAMs among different pairwise comparisons.

DAMs were identified and visualized using volcano plots (Figure 4A–D, Tables S3 and S4). A total of 2111 DAMs were identified across the four comparisons. Among them, 960 DAMs, including 327 upregulated and 633 downregulated metabolites, were identified in the BS vs. PD comparison, and 1124 DAMs, including 314 upregulated and 810 downregulated metabolites, were identified in WS vs. CS comparison. Among the four comparisons, the PD vs. YG group exhibited the highest number of differential metabolites, with 1244 DAMs, including 561 upregulated and 683 downregulated metabolites, whereas the CS vs. BS group showed fewer DAMs, with 882 metabolites, including 371 upregulated and 511 downregulated metabolites. These DAMs were mainly terpenoids and flavonoids, represented by compounds such as euscaphic acid, 2,3,23-trihydroxyolean-12-en-28-oic acid, epicatechin-3′-O-β-D-glucopyranoside, and trilobatin. These results strongly indicated significant metabolic reprogramming during fruit development, and the differential accumulation of key metabolites, such as terpenoids and flavonoids, provided insight into the molecular basis underlying fruit-quality formation and its dynamic changes.

Venn diagram analysis was performed to visualize the shared and unique DAMs among different pairwise comparisons (Figure 4E; Table S4). A total of 142 DAMs were commonly identified across all four comparisons, representing core metabolites that changed continuously throughout fruit development. In addition, 185, 99, 66, and 175 DAMs were uniquely identified in PD vs. YG, BS vs. PD, CS vs. BS, and WS vs. CS comparisons, respectively. The stage-specific DAMs may contribute to the characteristic metabolic features of each developmental transition.

### 3.5. Dynamic Changes and Functional Enrichment of DAMs During Fruit Development

**Figure 5:**
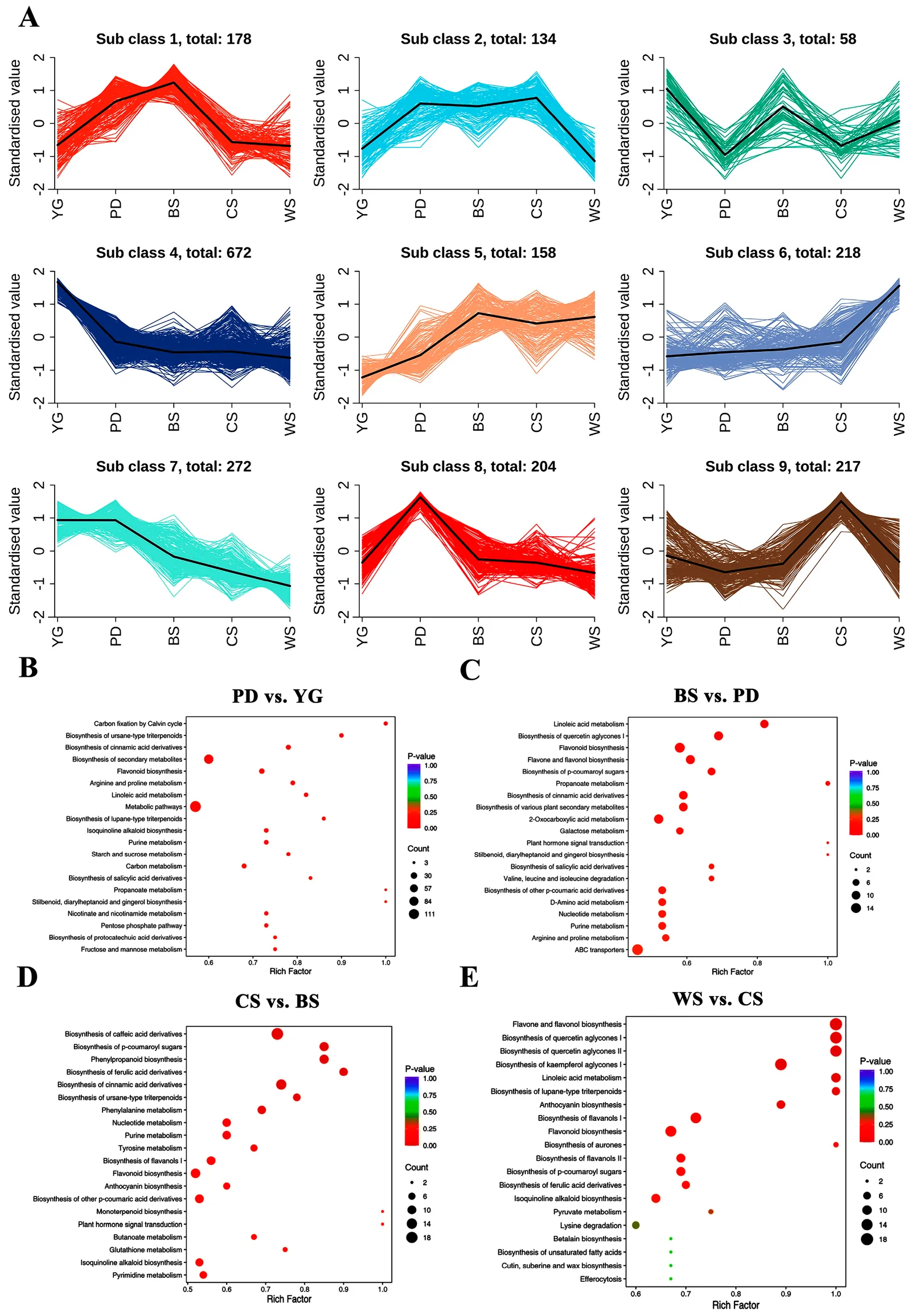
DAMs analysis of Junzao fruits at different developmental stages. (**A**) K-means clustering of DAMs. (**B**–**E**) Kyoto Encyclopedia of Genes and Genomes (KEGG) pathway enrichment analysis of DAMs for each pairwise comparison: (**B**) PD vs. YG, (**C**) BS vs. PD, (**D**) CS vs. BS, and (**E**) WS vs. CS. Dot color indicates the adjusted *p*-value, with darker red representing greater enrichment significance. Dot size represents the number of metabolites enriched in each pathway.

K-means clustering analysis was performed to characterize the dynamic accumulation patterns of DAMs across the five developmental stages (Figure 5A). Based on their temporal profiles, all DAMs were classified into nine distinct clusters (Class 1–9), each representing a unique accumulation pattern (Table S5). Class 4 and Class 7 metabolites showed a continuous decrease during development, whereas Class 6 metabolites exhibited low accumulation at early stages, followed by a sharp increase at the WS stage. Class 1, 2 and Class 8 metabolites reached peak abundance at the BS or PD stage and then gradually declined. Class 5 metabolites showed peak accumulation at the BS stage. Class 9 metabolites gradually increased from the YG to CS stages, followed by a decrease at the WS stage. Class 3 metabolites exhibited fluctuating patterns with multiple peaks. These diverse accumulation patterns reflected the complex metabolic regulation during of jujube fruit development.

KEGG pathway enrichment analysis was performed for the functional annotation and enrichment analysis of the differential metabolites, and the bubble plots illustrate the major biological pathways involving these metabolites (Figure 5B–E; Table S6). Notably, in the PD vs. YG group, DAMs were primarily enriched in ursane-type triterpenoids biosynthesis, cinnamic acid derivatives biosynthesis, and flavonoid biosynthesis pathways (*p* < 0.05). In the BS vs. PD comparison, DAMs were mainly enriched in Linoleic acid metabolism, quercetin aglycones biosynthesis, and flavonoid biosynthesis pathways (*p* < 0.05). In the CS vs. BS comparison, DAMs were mainly enriched in caffeic acid derivatives biosynthesis, phenylpropanoid biosynthesis, p-coumaroyl sugar biosynthesis, ferulic acid derivative biosynthesis, and ursane-type triterpenoid biosynthesis pathways (*p* < 0.05). In the WS vs. CS comparison, DAMs were mainly enriched in flavone and flavonol biosynthesis, quercetin and kaempferol aglycones biosynthesis, and lupane-type triterpenoids biosynthesis pathways (*p* < 0.05). These results suggested that flavonoid, triterpenoids and phenylpropanoid metabolism played crucial roles throughout fruit development.

### 3.6. Correlation Analysis of Secondary Metabolites in Junzao and Antioxidant Activity

**Figure 6:**
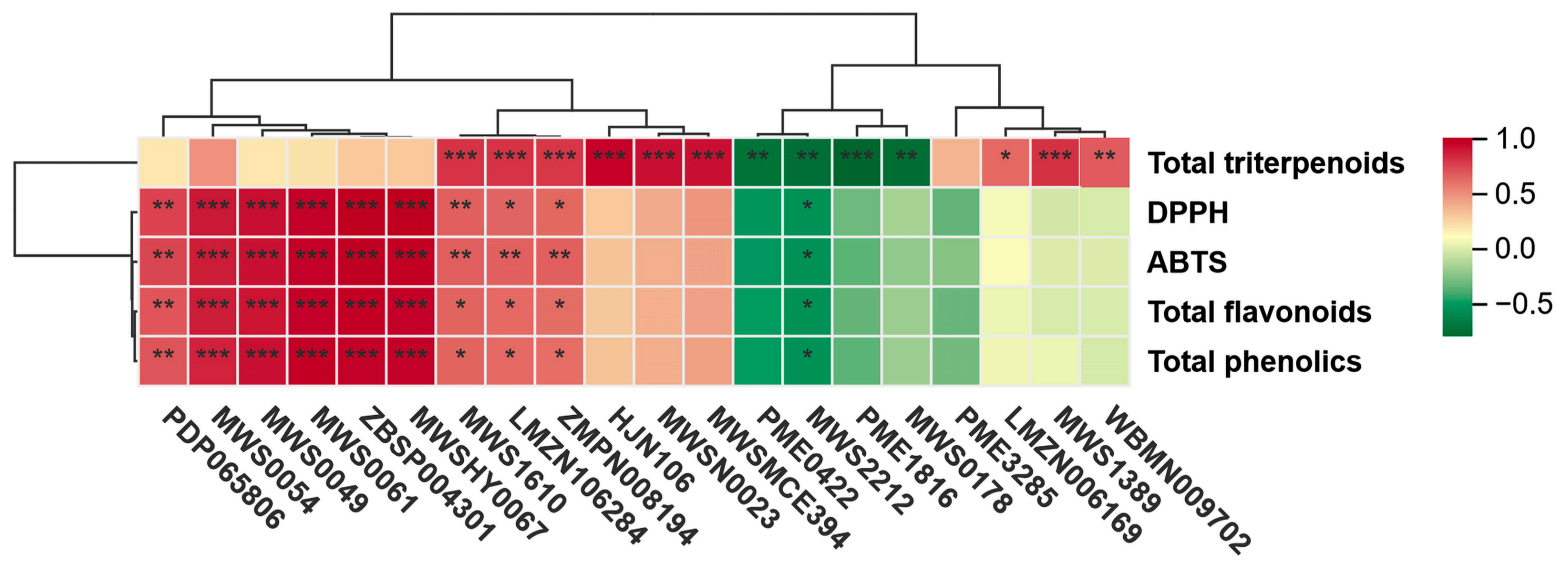
Heatmap of Spearman correlation analysis between antioxidant capacity and metabolites. Dendrograms above and at the left indicate hierarchical clustering of samples and traits, respectively. Asterisks inside cells denote the significance of pairwise comparisons: * *p* < 0.05, ** *p* < 0.01, *** *p* < 0.001. Red represents a positive correlation, and green represents a negative correlation.

To gain deeper insight into the key antioxidant components in Junzao, twenty metabolites were selected that showed trends consistent with those of total flavonoids, total phenolics, total triterpenes, DPPH, and ABTS for further Spearman correlation analysis (Figure 6, Table S7). A total of nine metabolites exhibited strong positive correlations with both antioxidant assays (|r| ≥ 0.5, *p* < 0.05), whereas one metabolite exhibited a strong negative correlation. These metabolites included six flavonoids: ZBSP004301 (quercetin-7-O-rutinoside), MWSHY0067 (rutin), MWS0061 (hyperin), MWS0049 ((+)-gallocatechin), MWS0054 ((+)-catechin), and PDP065806 (isoquercitrin); three triterpenes: LMZN106284 (alphitolic acid), MWS1610 (crataegolic acid), and ZMPN008194 (corosolic acid); and one phenolic acid: MWS2212 (caffeate). The above results indicate their substantial contribution to the antioxidant potential of Junzao fruit. These findings further highlight the important role of several metabolites, particularly flavonoids and triterpenes, in contributing to antioxidant activity during fruit development.

## 4. Discussion

The functional value of a medicine-food homologous fruit is not fixed but changes continuously during development, as carbon flux is redistributed among competing metabolic pathways [34]. In this study, widely targeted metabolomics was combined with in vitro antioxidant assays to characterize the temporal trajectory of secondary metabolites across five developmental stages of Junzao and to determine the stage at which antioxidant capacity is maximal. Our results demonstrate that the antioxidant potential of Junzao is concentrated in the immature, pre-veraison fruit rather than in the commercially harvested fully ripe fruit.

The traditional view that fully ripened fruits possess the highest nutritional and functional value has long influenced agricultural practices and product development [35]. However, our results revealed that Junzao fruits collected at the YG and BS stages exhibited markedly higher TPC (47.30 mg GAE/g DW), TFC (51.40 mg RE/g DW), TTC (56.86 mg OAE/g DW), and antioxidant activity (DPPH, 73.17%; ABTS, 95.07%) than those at the WS stage, with a clear monotonic decline during ripening. As the fruit enters veraison and full maturation, carbon flux may be progressively redirected from the phenylpropanoid and terpenoid pathways toward sugar metabolism and cell-wall modification, producing a dilution effect in which rapid gains in fresh weight outpace secondary-metabolite biosynthesis [36,37]. Similar trends in jujube species were reported by Yan et al. [38] and Hu et al. [39]. This “early-stage enrichment” phenomenon is not specific to jujube but aligns with a growing body of evidence across diverse fruit species. Similar enrichment of antioxidants and phenolics at early developmental stages has been documented in raspberry, cherry, and peach [40,41,42], in which bioactive compounds peak before maturity and decline during color transition and ripening. Collectively, these data highlight the high bioactive potential of immature fruits, reinforce the nonlinear relationship between developmental stage and bioactive value, and justify a re-evaluation of immature Junzao fruits as functional resources rather than agricultural by-products.

Fruit ripening is governed by complex transcriptional and metabolic reprogramming, which orchestrates the stage-dependent accumulation of secondary metabolites [43]. Widely targeted metabolomics in the present study identified 2388 secondary metabolites, of which 29 and 32 were uniquely detected at the YG and PD stages, respectively; moreover, 1244 DAMs were identified between PD and YG. KEGG enrichment further indicated that ursane-type triterpenoid biosynthesis, flavonoid biosynthesis, and cinnamic acid derivative biosynthesis pathways were highly active at early stages, whereas anthocyanin and lupane-type triterpenoid biosynthesis dominated at the WS stage. These observations are consistent with those of Tang et al. [44], who reported that PAL, CHS, and F3H transcript levels in winter jujube peaked at the young-fruit stage, and with the chemical–ecological paradigm in which immature fruits mobilize defensive metabolites to counter pathogen attack, herbivory, and UV stress [45,46]. Analogous temporal shifts in secondary metabolism have been observed in blueberry, tomato, and pepper, where phenylpropanoid and defensive pathways are most active before maturity and decline during ripening [47,48,49]. The stage-specific accumulation patterns observed in Junzao support the metabolic reprogramming paradigm and provide a high-resolution temporal atlas of bioactive compound dynamics, offering valuable insights for targeted harvesting and functional exploitation.

The antioxidant activity of Junzao fruits was highest at the YG stage, as evidenced by DPPH (73.17%) and ABTS (95.07%) radical-scavenging assays. Spearman correlation analyses identified a small set of metabolites correlated with both DPPH and ABTS activity (r ≥ 0.8, *p* < 0.01), dominated by flavonoids, including six compounds, and accompanied by triterpenes and one phenolic acid. The superior antioxidant activity observed in immature Junzao fruits may be attributed to their high levels of phenolics, flavonoids, and triterpenoids, which act through complementary mechanisms [50,51]. These findings position immature Junzao as a highly competitive natural-antioxidant source, comparable or even superior to other well-studied immature fruits, including immature apple [52], unripe blueberry [53], and unripe kiwifruit [54]. Three complementary mechanisms may explain this antioxidant advantage. Flavonoids such as rutin are suggested to protect against liver injury via inhibition of oxidative stress, inflammation, and apoptosis and via Nrf2 upregulation [55], whereas catechins function as indirect radical scavengers that bolster the endogenous antioxidant defense through regulating enzymatic activities and signaling cascades [56]. Triterpenoids, including oleanolic and crataegolic acid, have been reported to mitigate I/R-induced myocardial injury and oxidative stress by activating the Nrf2/ARE pathway and attenuating ROS generation through improved mitochondrial function and inhibition of NADPH oxidase [57,58]. Phenolic compounds, such as ferulic acid and caffeic acid, directly scavenge ROS, with their activity determined by hydroxyl substitution and conjugation [59]. Taken together, the superior antioxidant capacity of immature Junzao fruits likely arises from a coordinated interplay between direct radical scavenging and the induction of endogenous antioxidant defenses.

Traditionally, the jujube industry has focused primarily on harvesting fully ripe fruits, with limited utilization of immature fruits. This practice, which prioritizes fully ripe fruits for fresh consumption or processing, overlooks the considerable functional potential of earlier developmental stages. Based on our findings, a target-oriented precision harvesting model, in which immature fruits at the YG-BS stages are harvested for their high antioxidant activity and bioactive compound content, may be particularly suitable for functional-food and nutraceutical applications. This approach aligns with precision-agriculture principles, which prioritize harvest timing according to specific end-use objectives [60]. Given the pronounced astringency and sourness, as well as the limited palatability of immature fruits, we do not advocate their direct fresh consumption. Instead, immature fruits and agricultural by-products, such as thinning fruits and pruning residues, may be better positioned as raw materials for extraction and the development of standardized functional-food or nutraceutical ingredients, consistent with circular-economy and green-agriculture goals [61].

Although this study provides important insights into the temporal metabolome of Junzao fruits and their potential health benefits, several limitations and possible safety considerations should be acknowledged. First, only a single cultivar grown at one location was examined. Given that metabolic profiles can vary substantially among *Ziziphus* cultivars and growing environments, the generalizability of these findings should be tested through comparative multi-cultivar studies [62]. Second, antioxidant capacity was evaluated using two chemical assays (DPPH and ABTS), which measure the intrinsic electron- and hydrogen-atom-donating capacity of the extracts but do not capture cellular antioxidant responses, and take no account of gastrointestinal digestion, bioaccessibility, first-pass metabolism, or gut-microbiota-mediated biotransformation [63,64]. Third, immature fruits are markedly astringent and sour, and excessive intake of concentrated phenolic and triterpenoid preparations may exert pro-oxidant effects or interfere with nutrient absorption; effective and safe dose ranges therefore need to be defined before product development [65]. Future studies should therefore focus on: (i) cross-cultivar and cross-stage metabolic fingerprinting to identify optimal functional germplasm; (ii) pharmacodynamic and pharmacokinetic validation of immature Junzao extracts in cell-based and animal models of antioxidant activity, as well as assessment of the stability and bioaccessibility of the key antioxidants during processing and simulated digestion; and (iii) isolation, structural elucidation, and structure–activity analysis of key triterpenoids and flavonoids.

## 5. Conclusions

In this study, widely targeted UPLC-MS/MS metabolomics was combined with in vitro antioxidant assays to resolve the dynamic accumulation of secondary metabolites in Junzao fruits across five ripening stages. A total of 2388 secondary metabolites were identified, dominated by terpenoids and flavonoids, and 2111 differentially accumulated metabolites were distributed across nine distinct temporal patterns, reflecting a pronounced metabolic reprogramming from defense-related phenylpropanoid and triterpenoid metabolism in immature fruits toward ripening-associated metabolism in mature fruits. Immature fruits (YG-BS) accumulated markedly higher levels of phenolics, flavonoids, and triterpenoids and exhibited substantially stronger DPPH and ABTS radical-scavenging activities than fully ripe fruits. These findings provide a high-resolution temporal atlas of secondary metabolism in Junzao and indicate that the pre-veraison developmental window, rather than commercial maturity, is optimal when antioxidant capacity is the target attribute, thereby supporting stage-specific harvesting strategies and the valorization of immature fruits and thinning by-products as functional-food and natural-antioxidant raw materials. It should be noted that these conclusions are based on one cultivar and on chemical antioxidant assays; cellular and in vivo studies will be required to confirm their physiological relevance.

## Data Availability

The original contributions presented in this study are included in the article/Supplementary Materials. Further inquiries can be directed to the corresponding authors.

## Supplementary Materials

The following supporting information can be downloaded at: https://www.mdpi.com/article/10.3390/antiox15081038/s1, Figure S1: Overlaid the total ion current chromatograms of QC and samples in negative (A) and positive (B) ionization modes; Figure S2: (A–D) Permutation tests of orthogonal partial least squares discriminant analysis (OPLS-DA) models for pairwise comparisons: (A) PD (expansion stage) vs. YG (young-fruit stage), (B) BS (white-ripe stage) vs. PD, (C) CS (crispy-ripe stage) vs. BS, (D) WS (full-ripe stage) vs. CS; Table S1: All secondary metabolites identified in *Ziziphus jujuba* cv. ‘Junzao’ (Junzao) fruits during different growth stages; Table S2: Metabolites identified in Junzao fruits at five different growth stages; Table S3: Differentially accumulated metabolites (DAMs) screened in four pairwise comparisons; Table S4: Venn diagram results of DAMs screened from four pairwise comparisons; Table S5: The results of K-means analysis; Table S6: The results of Kyoto Encyclopedia of Genes and Genomes (KEGG) analysis (*p* < 0.05); Table S7: The results of heatmap analysis between antioxidant capacity and metabolites.
