# Supplementary material for: Widely Targeted Metabolomics Reveals Dynamic Secondary Metabolite Accumulation and Antioxidant Biomarkers Across Ripening Stages of Ziziphus jujuba cv. ‘Junzao’ Fruit
Source: Antioxidants (Basel). 2026 Aug 20;15(8):1038. doi: 10.3390/antiox15081038 (PMC13509210; doi:10.3390/antiox15081038)
Supplement: Supplementary file 1 [file antioxidants-15-01038-s001.zip › Supplementary Figure S1-S2.pdf]

Negative ion

(A)

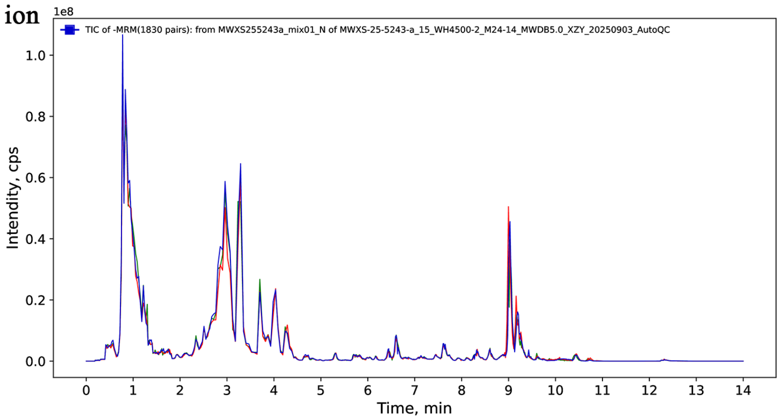

Positive ion

(B)

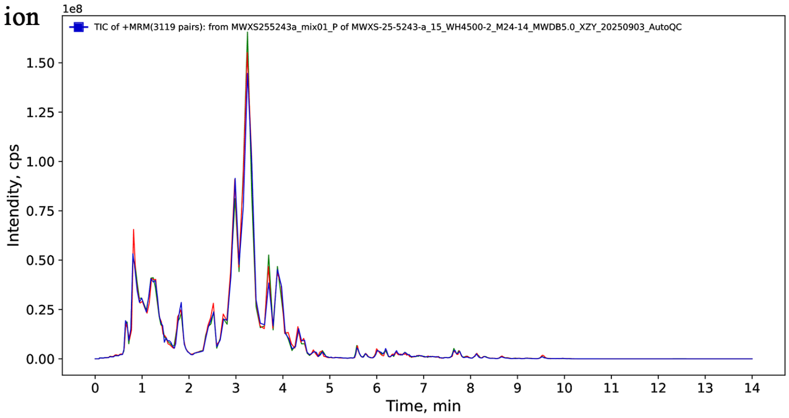

Figure S1 Overlaid the total ion current chromatograms of QC and samples in negative (A) and positive (B) ionization modes.

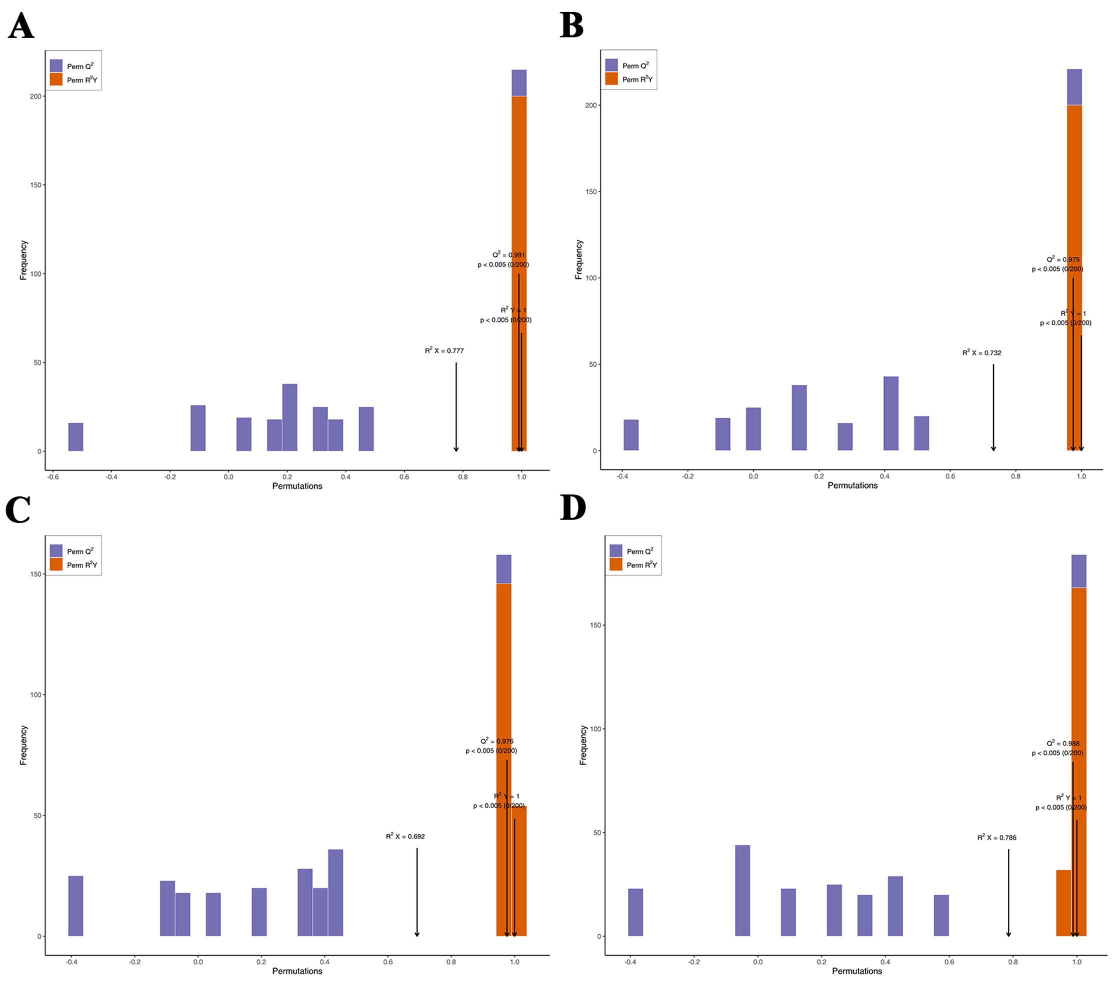

Figure S2(A–D) Permutation tests of orthogonal partial least squares discriminant analysis (OPLS-DA) models for pairwise comparisons: (A) PD (expansion stage) vs. YG (young-fruit stage), (B) BS (white-ripe stage) vs. PD, (C) CS (crispy-ripe stage) vs. BS, (D) WS (full-ripe stage) vs. CS.
